# The influence of dipeptidyl peptidase-4 inhibitor on the progression of type B intramural hematoma

**DOI:** 10.3389/fcvm.2022.969357

**Published:** 2022-10-18

**Authors:** Qu Chen, Dandan Jiang, Zhonggui Shan

**Affiliations:** ^1^Department of Cardiovascular Surgery, The First Affiliated Hospital of Xiamen University, School of Medicine, Xiamen University, Xiamen, Fujian, China; ^2^Department of Respiratory Medicine, Xinglin Branch of the First Affiliated Hospital of Xiamen University, School of Medicine, Xiamen University, Xiamen, Fujian, China

**Keywords:** diabetes mellitus, dipeptidyl peptidase-4 inhibitor, intramural hematoma, outcome, eosinophil

## Abstract

**Objectives:**

Investigating whether dipeptidyl peptidase-4 inhibitors (DPP4i) could influence the progression of type B intramural hematoma (IMHB) in patients with diabetes mellitus (DM).

**Materials and methods:**

Uncomplicated IMHB patients were matched by age, sex, and body mass index. Cox proportional hazard models were constructed to identify risk factors. A Kaplan–Meier survival analysis was used to estimate all-cause and aorta-related mortality.

**Results:**

Ninety-six matched IMHB patients were divided into Group A (*n* = 32, IMHB patients without DM), Group B (*n* = 32, IMHB patients with DMreceiving oral antidiabetic drugs [without DPP4i]) and Group C (*n* = 32, IMHB patients with DM receiving oral antidiabetic drugs [with DPP4i]). Group C had the lowest rate of aorta-related adverse events (3.1%), aorta-related mortality (0.0%) and reintervention (3.1%). Cox proportional hazard models revealed that a lower eosinophil count (per 0.1, HR, 0.48; 95% CI, 0.29–0.79, *P* = 0.004) and a higher neutrophil to lymphocyte ratio (NLR) (HR, 1.13; 95% CI, 1.05–1.21, *P* = 0.001) were associated with higher occurrences of aorta-related adverse events. A lower eosinophil count (per 0.1, HR, 0.40; 95% CI, 0.18–0.89, *P* = 0.025) and a higher NLR (HR, 1.19; 95% CI, 1.08–1.32, *P* = 0.001) were also associated with increased aorta-related mortality.

**Conclusion:**

DPP4i administration in DM patients with IMHB was associated with lower aorta-related mortality and more benign progression than in those who did not receive DPP4i or those without DM. Furthermore, a higher eosinophil count and a lower NLR ratio are potential protective factors that may explain the potential therapeutic benefit of DPP4i.

## Introduction

Studies have found an inverse relationship between diabetes mellitus (DM) and the prevalence of aortic diseases (1–3). However, other studies indicate that the protective effect of hyperglycemia in preventing the aortic aneurysm development process could be diminished by insulin treatment (4) and that DM results in impaired activation of the protective anti-inflammatory pathway in vascular inflammation (5). In addition, long-lasting clinical hyperglycemia (>10 years), but not prediabetes, independently played an important role in reducing abdominal aortic aneurysm risk (6). One possible explanation for the controversial roles of DM in aortic disease is that the protective effects of DM are due to other factors, such as long-term administration of oral antidiabetic drugs, which may slow the progression of aortic disease.

Since 2006, dipeptidyl peptidase-4 inhibitors (DPP4i) have been approved for use in type 2 DM, and currently, the vascular protective roles of DPP4i have been described (7). Kohashi et al. (8) and Takahara et al. (9) both indicated that DPP4i could suppress macrophage infiltration and abdominal aortic aneurysm formation. Our previous studies on the influence of DM on clinical outcomes in intramural hematoma (IMH) patients also revealed an association between DM and better clinical outcomes and lower inflammatory biomarkers (10, 11). DPP4i administration may result in higher concentrations of the chemokine eotaxin-1 and enhance the recruitment of eosinophils to the vascular lesion (12). Moreover, the eosinophils could release chemokines to regulate macrophage and monocyte polarization, and block inflammatory activation in the aorta (13). This may also trigger the normalization of vascular function such as restoring physiological perfusion, maintaining normal oxygenation, and enhancing the angiogenesis process (14).

In Asian countries, the “wait-and-watch strategy” is the first line treatment for type B IMH (IMHB) patients (15, 16). Whether DPP4i administration could influence the clinical outcomes of IMHB is unclear because using DPP4i alone does not exist in clinical practice. Therefore, in this retrospective study, we compared the clinical outcomes (aortic remodeling, clinical complications, and all-cause mortality) in IMHB patients with and without type 2 DM. In addition, we also aimed to clarify the potential association between the DPP4i administration and IMHB patient clinical outcomes.

## Materials and methods

### Investigated subject and survey method

From January 2007 to December 2020, 395 IMHB patients who received at least 2 weeks of the “wait and watch strategy” (controlling pain, heart rate, and blood pressure, receiving serial imaging, and necessary thoracic endovascular aortic repair [TEVAR]/open surgery) (15) were included in this study. These patients were matched by age, sex, and BMI, which are well-known risk factors for aortic diseases (15, 16) and DM (1). Finally, only 96 cases were included in the new cohort (32 cases in each group). Data were obtained via telephone follow-up by a cardiac surgery department nursing team. The time-points chosen for completion were: preoperative intervention, 6 weeks, 3, 6, and 12 months postintervention, and then annually. At the time of data collection, a questionnaire (Supplementary material 1) had to be fully completed, and the dataset included the laboratory test results, computed tomography angiography (CTA) examination, chronic disease history and management, and IMHB progression (Supplementary material 1). This study was performed in accordance with the principles of the Declaration of Helsinki and the study protocol was approved by the Institutional Ethical Committee of Xiamen University (Xiamen, China). The approval number is XUEC20202097. Because of the retrospective nature of the study, the requirement for patient consent for inclusion was waived.

**FIGURE 1:**
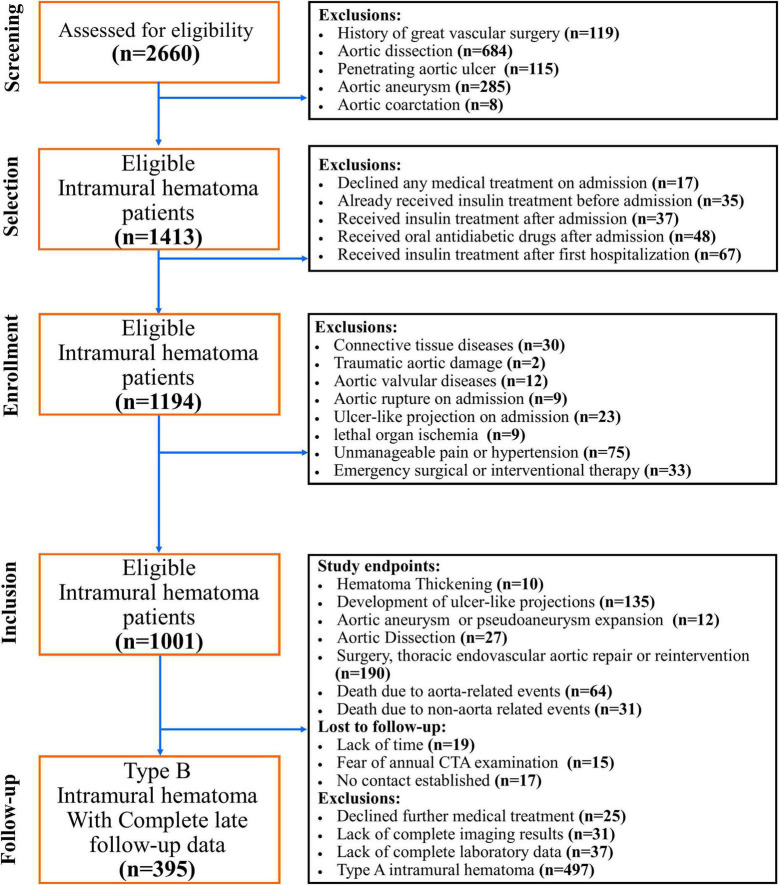
CONSORT diagram. This graphic illustrates the CONSORT diagram of the patient selection process. From January 2007 to December 2020, a total of 96 matched IMHB patients who received the “wait-and-watch strategy” in a single institution were included in this study. Patients who declined medical care, those who did not have CTA images to assess the progression of IMHB, those who did not have complete laboratory data, and those who could not be communicated for follow-up were all considered to be missing data.

To determined the impact of oral antidiabetic drugs, especially DPP4i, type 2 DM patients who underwent insulin treatment before the onset of IMHB and newly diagnosed type 2 DM patients who underwent long-term insulin treatment after the onset of IMHB were excluded. In addition, patients who had traumatic aortic damage, aortic connective tissue illnesses, or aortic valvular diseases were not allowed to participate in the study. Patients who had aortic connective tissue diseases detected through pathology or genetic testing. Aortic rupture, ulcer-like projection (ULP) development on admission, potentially deadly organ ischemia, unmanageable pain/hypertension, and emergency surgical or interventional therapy on admission were not included in this study. Patients who declined medical treatment, patients who did not have CTA pictures to assess the progression of IMHB, patients who did not have complete laboratory data, and patients who could not be located for follow-up were all considered to be missing data (Figure 1).

The primary outcome was to estimate all-cause mortality under different treatment strategies. Secondary outcomes were aimed to evaluate the influence of different treatment strategies on the aorta-related mortality and aorta-related adverse events that required surgery/TEVAR.

### Blood glucose management

The following are some of the diagnostic criteria for type 2 DM: hemoglobin A1c [HbA1c] ≥ 6.5%, fasting plasma glucose ≥126 mg/dl, and 2 h plasma glucose ≥200 mg/dl (17). During the perioperative period, the insulin pump with short-acting insulin was used to achieve rapid glucose control during the stay in the intensive care unit (with the help of a physician, DJ), and then the patients were transferred to oral antidiabetic medications. Postprandial blood glucose levels should be less than 180 mg/dl, and proper fasting and premeal blood glucose levels should be between 80 and 130 mg/dl. The goal blood glucose level includes these ranges (17, 18). The HbA1c level was measured every 3 months to determine whether glycemic targets had been met and maintained, and those who needed insulin treatment were excluded from the study.

### Statistical analysis

To compare categorical variables, Pearson’s chi-square test and Fisher’s exact test were employed. When necessary, we used Student’s *t*-test, Welch’s *t*-test, and the Mann–Whitney *U* test to compare continuous data. The Kolmogorov–Smirnov test was carried out to determine whether the data were normal. Both univariate and multivariate logistic regression analyses were utilized to discover independent risk variables that were linked to the occurrence of adverse outcomes that were associated with the aorta. During the course of the study, Cox proportional hazard models were utilized to evaluate the factors that were connected to aorta-related adverse events as well as aorta-related mortality. A Kaplan–Meier survival analysis with the log-rank test was used to assess the occurrence of aorta-related adverse events and aorta-related mortality. In the multivariable analysis, we only considered the factors that had a *P* value that was lower than 0.20 in the univariate analysis. IBM SPSS Statistics for Windows Version 26.0 (IBM Corp., Armonk, NY) was used to conduct statistical analysis. Comparisons with a *P* value less than 0.005 were considered statistically significant difference.

## Results

### Patient characteristics

From January 2007 to December 2020, 96 matched IMHB patients who received the “wait-and-watch strategy” in a single institution were included in this study. These patients were divided into three groups: Group A (*n* = 32, IMHB patients without DM), Group B (*n* = 32, IMHB patients with type 2 DM and receiving oral antidiabetic drugs [without DPP4i at admission]) and Group C (*n* = 32, IMH patients with type 2 DM receiving oral antidiabetic drugs [with DPP4i at admission]). These three groups were significantly different from one another in a number of covariables (including known risk factors for aortic diseases and known factors that could influence the progression and long-term outcomes of aortic diseases; see Table 1). Group B and Group C had significantly higher incidence rates of dyslipidemia than Group A. Regarding the laboratory test results, compared with the other two groups, Group C had the lowest counts of white blood cells and neutrophils, the lowest neutrophil-to-lymphocyte ratio, and the lowest levels of C-reactive protein and D-dimer (Table 1 and Supplementary material 2). Group C also had the highest counts of lymphocytes and eosinophils among the three groups, and the pairwise comparison results also demonstrated significant differences among the three groups (Supplementary material 2). Interestingly, Group C had the largest ascending aortic diameter compared to Groups A and B, and a large ascending aortic diameter (>4.0 cm) has been widely accepted as a risk factor for the lethal progression of aortic diseases (19). The descending aortic diameter in Group B and C was also significantly larger than that in Group A. However, the maximum hematoma thickness, another well-known predictor of intramural hematoma lethal progression (20), was smaller in Group C, and Group A had the thickest hematoma thickness compared to the other two groups (Table 1 and Supplementary material 2).

**TABLE 1:** Baseline characteristics^a^.

Variables	Group A (*n* = 32)	Group B (*n* = 32)	Group C (*n* = 32)	*P* value
Age, years	63.8 ± 10.3	63.8 ± 10.3	63.8 ± 10.3	1.000^b^
BMI, kg/m^2^	25.3 ± 2.3	26.3 ± 2.7	26.1 ± 1.8	0.270^b^
Gender, sex				1.000
Male, *n* (%)	17 (53.1%)	17 (53.1%)	17 (53.1%)	
Female, *n* (%)	15 (46.9%)	15 (46.9%)	15 (46.9%)	
Systolic pressure, mmHg	152 ± 21	156 ± 22	152 ± 22	0.592
Diastolic pressure, mmHg	83 ± 12	87 ± 14	84 ± 12	0.265
Heart rate, bpm	82 ± 15	84 ± 14	85 ± 16	0.650
Waist circumference, cm	82 ± 7	88 ± 10	87 ± 12	0.094
Exercise, *n* (%)	3 (9.4%)	4 (12.5%)	1 (3.1%)	0.385
Concomitant diseases				
Smoking, *n* (%)	24 (75.0%)	124 (75.0%)	21 (65.6%)	0.629
Drinking, *n* (%)	23 (71.9%)	26 (81.3%)	22 (68.8%)	0.495
Hypertension, *n* (%)	24 (75.0%)	26 (81.3%)	26 (81.3%)	0.777
Dyslipidemia, *n* (%)	15 (46.9%)	29 (90.6%)	27 (84.4%)	<0.001
Metabolic syndrome	0 (0%)	2 (6.3%)	3 (9.4%)	0.228
Gout, *n* (%)	2 (6.3%)	5 (15.6%)	8 (25.0%)	0.118
Hypothyroidism, *n* (%)	1 (3.1%)	3 (9.4%)	5 (15.6%)	0.230
Anemias, *n* (%)	1 (3.1%)	1 (3.1%)	0 (0%)	0.600
Coronary heart disease, *n* (%)	7 (21.9%)	13 (40.6%)	8 (25.0%)	0.210
Atrial fibrillation, *n* (%)	5 (15.6%)	6 (18.8%)	6 (18.8%)	0.931
Heart failure, *n* (%)	2 (6.3%)	4 (12.5%)	5 (15.6%)	0.487
Obstructive Sleep Apnea, *n* (%)	22 (68.8%)	26 (81.3%)	20 (62.5%)	0.244
Chronic obstructive pulmonary disease, *n* (%)	2 (6.3%)	1 (3.1%)	3 (9.4%)	0.587
Asthma, *n* (%)	1 (3.1%)	1 (3.1%)	2 (6.3%)	0.770
Renal failure, *n* (%)	1 (3.1%)	0 (0.0%)	1 (3.1%)	0.600
Dialysis	1 (3.1%)	0 (0.0%)	1 (3.1%)	0.600
Stroke, *n* (%)	0 (0.0%)	0 (0.0%)	1 (3.1%)	0.364
Family history of stroke, *n* (%)	0 (0.0%)	0 (0.0%)	1 (3.1%)	0.364
Peripheral ischemia, *n* (%)	2 (6.3%)	3 (3.1%)	2 (6.3%)	0.857
Carotid artery disease, *n* (%)	6 (18.8%)	12 (37.5%)	13 (40.6%)	0.129
Liver steatosis, *n* (%)	10 (31.3%)	26 (81.3%)	27 (84.4%)	<0.001
Liver cirrhosis, *n* (%)	1 (3.1%)	0 (0.0%)	0 (0.0%)	0.364
Gastrointestinal bleeding history, *n* (%)	0 (0.0%)	0 (0.0%)	0 (0.0%)	–
Depression, *n* (%)	0 (0.0%)	0 (0.0%)	0 (0.0%)	–
History of cancer, *n* (%)	1 (3.1%)	2 (6.3%)	2 (6.3%)	0.810
Drug abuse, *n* (%)	0 (0.0%)	0 (0.0%)	0 (0.0%)	–
Antihypertensive medications				
ACEI/ARB, *n* (%)	30 (93.8%)	31 (96.9%)	28 (87.5%)	0.340
β-blockers, *n* (%)	28 (87.5%)	31 (96.9%)	27 (84.4%)	0.234
Calcium antagonists, *n* (%)	15 (46.9%)	15 (46.9%)	15 (46.9%)	1.000
Diuretic, *n* (%)	7 (21.9%)	10 (31.3%)	14 (43.8%)	0.172
Urapidil, *n* (%)	25 (78.1%)	27 (84.4%)	29 (90.6%)	0.387
Nitrates, *n* (%)	7 (21.9%)	12 (37.5%)	8 (25.0%)	0.339
Laboratory test on admission				
White blood cell (10^9/L)	12.0 ± 1.7	11.4 ± 2.1	8.8 ± 1.5	<0.001^b^
Neutrophils (10^9/L)	10.8 ± 1.6	9.7 ± 2.0	6.7 ± 1.5	<0.001^b^
Lymphocyte (10^9/L)	0.82 ± 0.21	1.25 ± 0.34	1.52 ± 0.25	<0.001^b^
Eosinophils (10^9/L)	0.054 ± 0.033	0.105 ± 0.068	0.294 ± 0.029	<0.001^b^
Monocytes (10^9/L)	0.319 ± 0.138	0.373 ± 0.149	0.336 ± 0.151	0.338
Platelet (10^9/L)	224.6 ± 65.0	236.7 ± 61.8	254.3 ± 67.5	0.169
C-reactive protein (mg/L)	34.3 ± 14.4	15.8 ± 5.3	15.8 ± 5.5	<0.001^b^
D-dimer (ug/ml)	3.7 ± 0.5	2.9 ± 0.7	1.5 ± 0.5	<0.001^b^
Fasting plasma glucose, mg/dl	97 ± 9	165 ± 22	168 ± 20	<0.001
Hemoglobin, g/dl	128 ± 12	127 ± 12	129 ± 9	0.990
Cholesterol, mmol/L	5.4 ± 0.9	5.8 ± 0.7	5.8 ± 0.6	0.166
Triglyceride, mmol/L	1.8 ± 0.2	1.7 ± 0.5	1.7 ± 0.4	0.595
LDL, mmol/L	3.3 ± 0.5	3.3 ± 0.5	3.4 ± 0.5	0.947
VLDL, mmol/L	0.62 ± 0.24	0.60 ± 0.23	0.59 ± 0.22	0.873
HDL, mmol/L	1.20 ± 0.18	1.18 ± 0.17	0.12 ± 0.17	0.152
Lipid ratio	2.84 ± 0.54	2.89 ± 0.72	3.04 ± 0.55	0.270
Non-HDL cholesterol, mmol/L	3.95 ± 0.52	3.92 ± 0.58	3.96 ± 0.47	0.990
Geometric measurements				
Diameter of ascending aorta (mm)	38.0 ± 2.7	40.5 ± 2.6	41.3 ± 2.9	<0.001^b^
Diameter of descending aorta (mm)	28.7 ± 3.1	31.4 ± 3.7	32.2 ± 3.6	<0.001^b^
Hematoma thickness (mm)	7.9 ± 1.0	7.3 ± 0.9	6.5 ± 0.9	<0.001^b^

ACEI, angiotensin-converting enzyme inhibitor; ARB, angiotensin II receptor blocker; BMI, body mass index; HDL, high density lipoprotein; LDL, low density lipoprotein; VLDL, very low density lipoprotein. ^a^If quantitative variables are normally distributed, they are presented as mean ± standard deviations, and if they are abnormally distributed, they are presented as median (25th percentile, 75th percentile). Choose the appropriate statistical method based on the results of the normality test (Kolmogorov–Smirnov test) and the homogeneity of variances (Levene’s test). And the details of the questionnaire are presented in Supplementary material 1. ^b^Supplementary material 2 contains the details of the independent-samples Kruskal–Wallis test and pairwise comparisons of three groups.

### Disease progression, treatment, clinical outcomes, and late follow-up

The details of disease progression, treatment, clinical outcomes, and late follow-up results are summarized in Table 2. The occurrence rate of aorta-related adverse events during the acute phase was markedly higher in Group A (34.4%) and Group C had the lowest occurrence rate (3.1%) compared to Groups A and B (Table 2). The most common aorta-related adverse event was the development of ULP. Up to 28.1% of patients in Group A suffered from the development of ULP, and Group C had a comparatively lower rate of ULP development (3.1%). Compared to Group A and Froup B, fewer patients in Group C received surgery/TEVAR treatment during the acute phase (0 and 3.1%), and the mortality after the acute phase surgery/TEVAR was similar among the three groups (0%) (more details in Table 2).

**TABLE 2:** Disease progressions, treatments and outcomes.

Variables	Group A (*n* = 32)	Group B (*n* = 32)	Group C (*n* = 32)	*P* value
Aorta-related adverse events during the acute phase, *n* (%)	11 (34.4%)	8 (8.3%)	1 (3.1%)	0.007^c^
Hematoma thickening (thickness ≥ 10 mm), *n* (%)	1 (3.1%)	1 (3.1%)	0 (0.0%)	0.600
Development of ULPs, *n* (%)	9 (28.1%)	6 (18.8%)	1 (3.1%)	0.025^c^
Development of aortic dissection, *n* (%)	1 (3.1%)	1 (3.1%)	0 (0.0%)	0.600
Aortic aneurysm/Pseudoaneurysm development, *n* (%)	0 (0.0%)	0 (0.0%)	0 (0.0%)	–
Treatment				
Surgery, *n* (%)	1 (3.1%)	1 (1.0%)	0 (0.0%)	0.600
TEVAR^a^, *n* (%)	10 (31.3%)	7 (21.9%)	1 (3.1%)	0.013^c^
Died after surgery/TEVAR, *n* (%)	0 (0.0%)	0 (0.0%)	0 (0.0%)	–
Late follow-up				**–**
Median follow-up time (months)	56.0 (49.9–62.1)	44.0 (33.7–54.3)	49.0 (42.5–55.5)	**–**
Stable/Resolution of hematoma, *n* (%)	19 (59.4%)	24 (75.0%)	31 (96.9%)	0.002^c^
Aorta-related adverse events during the follow-up period, *n* (%)	13 (40.6%)	8 (25.0%)	1 (3.1%)	0.002^c^
Hematoma thickening (thickness ≥ 10 mm), *n* (%)	0 (0.0%)	0 (0.0%)	0 (0.0%)	–
Development of ULPs, *n* (%)	9 (28.1%)	5 (15.6%)	1 (3.1%)	0.023^c^
Development of aortic dissection, *n* (%)	2 (6.2%)	3 (9.4%)	0 (0.0%)	0.228
Aortic aneurysm/Pseudoaneurysm, *n* (%)	2 (6.2%)	1 (3.1%)	0 (0.0%)	0.356
Reintervention^b^, *n* (%)	13 (40.6%)	8 (25.0%)	1 (3.1%)	0.002^c^
All-cause death cases, *n* (%)	10 (31.3%)	4 (12.5%)	1 (3.1%)	0.007^c^
Aorta-related death cases, *n* (%)	8 (25.0%)	3 (9.4%)	0 (0.0%)	0.007^c^
Non-aorta-related death case, *n* (%)	2 (6.2%)	1 (3.1%)	1 (3.1%)	0.600

TEVAR, thoracic endovascular aortic repair; ULP: Ulcer-like projection. ^a^Valiant (Medtronic, Inc., Minneapolis, MN); Ankura (Lifetechmed, Inc., Shenzhen, China). ^b^Including TEVAR or open surgery. ^c^The results of the Z-test that compared each column proportions among different groups and calculated the adjusted *p*-value (Bonferroni method) are presented in the Supplementary material 3.

After discharge, 96 patients participated in the late follow-up, and the median follow-up times for each group were: 56.0 months for Group A (95% confidence interval [CI], 49.9–62.1 months), 44.0 months for Group B (95% CI, 33.7–54.3 months) and 49.0 months for Group C (95% CI, 42.5–55.5 months). A total of 96.9% of patients in Group C had a resolution of the hematoma or stable hematoma, which was dramatically higher than in Group A and Group B. The occurrence rate of aorta-related adverse events was up to 40.6% in Group A and 25.0% in Group B. The development of the ULP was still the most common aorta-related adverse event and affected 28.1% of the patients in Group A, which was significantly higher than that in Group B and group C (Table 2). Group A also had a greater reintervention rate during the follow-up period than the other two groups (40.6%). Compared with Group B, the reintervention rate in Group C was significantly lower (25.0 vs. 3.1%, Table 2).

**FIGURE 2:**
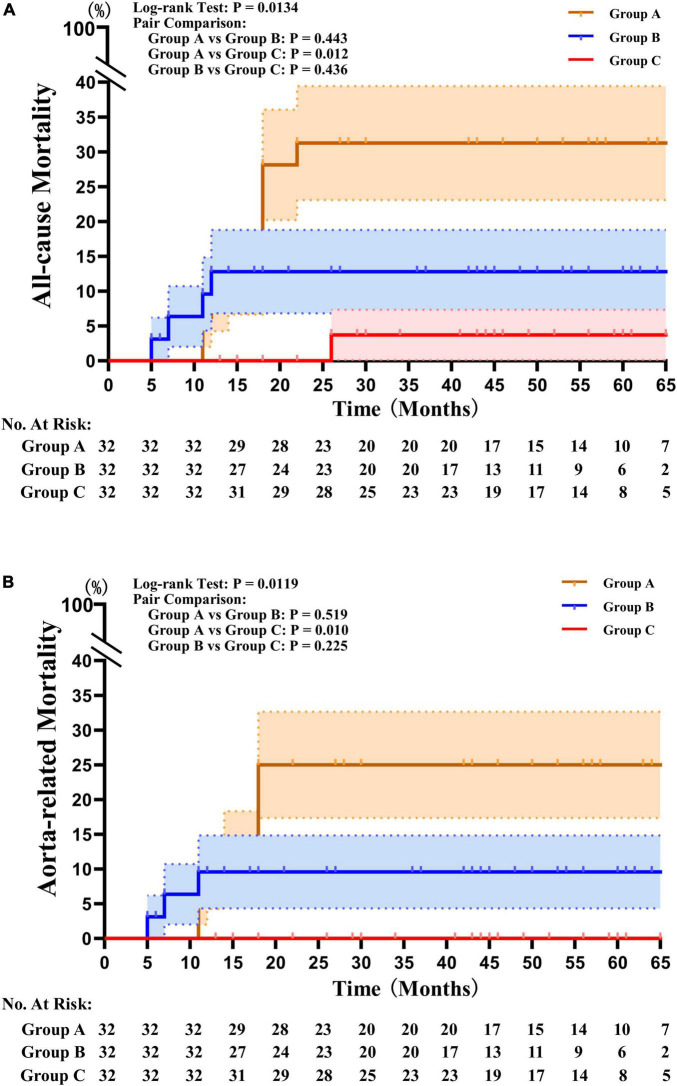
Kaplan–Meier survival analysis results. **(A)** Kaplan–Meier survival analysis revealed that the mortality rates from all causes were significantly lower in Group C than in Groups A and B (*P* = 0.0134). **(B)** The Kaplan–Meier survival analysis found that the mortality rates associated with the aorta were significantly lower in Group C than they were in Groups A and B (*P* = 0.0119).

There were significant differences in all-cause and aorta-related mortality among the three groups during the follow-up period. Group C had significantly lower all-cause mortality (3.1%) and aorta related mortality (0.0%) than Group A and Group B, and there was no a significant difference between Group A and Group B (Table 2). The Kaplan–Meier survival analysis revealed a significant decrease in the all-cause (*P* = 0.0134) and aorta-related mortality rates (*P* = 0.0119) in Group C compared with those in Group B and C (Figure 2).

### Logistic regression analyses and cox proportional hazard model

Logistic regression analyses indicated that a lower eosinophil count (per 0.1, odds ratio [OR], 0.24; 95% CI, 0.11–0.53, *P* < 0.001) and larger descending aortic diameters (OR, 1.26; 95% CI, 1.06–1.50, *P* = 0.008) were found to be significantly associated with aorta-related adverse events during the acute phase (Table 3). Cox proportional hazard models revealed that a lower eosinophil count (per 0.1, hazard ratio [HR], 0.48; 95% CI, 0.29–0.79, *P* = 0.004) and a higher neutrophil to lymphocyte ratio (NLR) (HR, 1.13; 95% CI, 1.05–1.21, *P* = 0.001) were associated with a higher occurrence rate of aorta-related adverse events during the follow-up period. A lower eosinophil count (per 0.1, HR, 0.40; 95% CI, 0.18–0.89, *P* = 0.025) and a higher NLR ratio (HR, 1.19; 95% CI, 1.08–1.32, *P* = 0.001) were significantly associated with aorta-related mortality during the follow-up period (see more details in Table 3).

**TABLE 3:** Variables associated with aorta-related adverse events and aorta-related mortality^ab^.

(A) Logistic regression analyses: significant predictors for aorta-related adverse events in the acute phase						
Variables	Univariate analysis				Adjusted multivariable analysis	
	Unadjusted OR (95% CI)	*P* value	Adjusted OR (95% CI)	*P* value	OR (95% CI)	*P* value
Neutrophil to lymphocyte ratio	0.89 (0.73–1.08)	0.226	0.89 (0.73–1.10)	0.303	–	–
Eosinophils per 0.1 (10^9/L)	0.35 (0.13–0.95)	0.039	0.29 (0.09–0.90)	0.032	0.24 (0.11–0.53)	<0.001
C-reactive protein on admission (mg/L)	1.04 (0.97–1.11)	0.269	1.04 (0.97–1.12)	0.299	–	–
D-dimer on admission (ug/ml)	1.38 (0.43–4.45)	0.594	1.26 (0.35–4.52)	0.719	–	–
Maximum ascending aorta diameter, mm	0.88 (0.66–1.49)	0.368	0.93 (0.68–1.27)	0.639	–	–
Maximum descending aorta diameter, mm	1.21 (0.98–1.49)	0.073	1.18 (0.94–1.50)	0.157	1.26 (1.06–1.50)	0.008
Maximum hematoma thickness, mm	1.22 (0.70–2.14)	0.487	1.05 (0.54–2.04)	0.893	–	–

**(B) Cox proportional hazard models: significant variables associated with aorta-related adverse events during the follow-up period**						

**Variables**	**Univariate analysis**				**Adjusted multivariable analysis**	
	**Unadjusted HR (95% CI)**	***P* value**	**Adjusted HR (95% CI)**	***P* value**	**HR (95% CI)**	***P* value**

Neutrophil to lymphocyte ratio	1.08 (0.97–1.20)	0.184	1.07 (1.06–1.21)	0.138	1.13 (1.05–1.21)	0.001
Eosinophils per 0.1 (10^9/L)	0.47 (0.21–0.97)	0.071	0.52 (0.22–0.97)	0.139	0.48 (0.29–0.79)	0.004
C-reactive protein on admission (mg/L)	1.01 (0.96–1.06)	0.781	1.01 (0.96–1.06)	0.773	–	–
D-dimer on admission (ug/ml)	0.71 (0.24–2.06)	0.527	0.76 (0.26–2.25)	0.623	–	–
Maximum ascending aorta diameter, mm	1.09 (0.89–1.32)	0.420	1.10 (0.88–1.36)	0.407	–	–
Maximum descending aorta diameter, mm	0.99 (0.84–1.17)	0.914	0.98 (0.81–1.17)	0.801	–	–
Maximum hematoma thickness, mm	1.19 (0.73–1.92)	0.486	1.25 (0.72–2.17)	0.437	–	–

**(C) Cox proportional hazard models: significant variables associated with aorta-related mortality during the follow-up period**						

**Variables**	**Univariate analysis**				**Adjusted multivariable analysis**	
	**Unadjusted HR (95% CI)**	***P* value**	**Adjusted HR (95% CI)**	***P* value**	**HR (95% CI)**	***P* value**

Neutrophil to lymphocyte ratio	1.17 (1.00–1.37)	0.056	1.28 (1.03–1.59)	0.028	1.19 (1.08–1.32)	0.001
Eosinophils per 0.1 (10^9/L)	0.41 (0.18–0.90)	0.026	0.46 (0.20–0.96)	0.070	0.40 (0.18–0.89)	0.025
C-reactive protein on admission (mg/L)	1.06 (0.96–1.16)	0.262	1.06 (0.96–1.18)	0.265	–	–
D-dimer on admission (ug/ml)	0.36 (0.057–1.96)	0.225	0.27 (0.35–2.13)	0.215	–	–
Maximum ascending aorta diameter, mm	1.08 (0.78–1.50)	0.653	1.22 (0.79–1.88)	0.367	–	–
Maximum descending aorta diameter, mm	1.00 (0.75–1.33)	0.982	1.01 (0.70–1.45)	0.965		
Maximum hematoma thickness, mm	0.97 (0.50–1.89)	0.925	1.25 (0.55–2.85)	0.597	–	–

CI: confidence interval; HR: hazard ration; OR: odds ratio. ^a^The adjusted logistic regression analyses and Cox proportional hazard models were based on presence of age, gender, body mass index, hypertension, dyslipidemia, coronary heart disease, obstructive sleep apnea and antihypertension therapy. ^b^The collinearity test was performed to check the collinearity of variables in the model.

## Discussion

The findings in this manuscript have three main implications for current clinical practice. First, DPP4i administration in IMHB patients with DM significantly improves disease progression compared to that in patients without DPP4i use or without DM. Previous IMHB treatment guidelines have emphasized the use of antihypertensive, heart rate-controlling, and analgesic drugs, and there have been no reports of DPP4i use (15). When compared to other oral hypoglycemic drugs, the biggest benefit of DPP4i is that it does not cause symptoms of hypoglycemia after use (17, 18). This makes it comparatively safer to use DPP4i alone than other oral anti-diabetic drugs, but there are currently no reports of the direct use of DPP4i to influence the progression of IMHB.

Second, the application of DPP4i appears to reduce the NLR and results in a lower level of D-dimer in IMHB patients and a higher eosinophil count. A lower NLR and a higher eosinophil count also better predict IMHB progression, which provides new predictive factors for the acute phase progression of IMHB. IMHB is generally considered to be acceptable for drug treatment and the “wait and watch technique” is the primary treatment option for patients in Asian nations who have IMHB (15, 16). However, the progression of IMHB is unpredictable, ranging from complete resolution to abrupt rupture (15, 21), although numerous predictors of IMHB evolution, such as maximum aortic diameter (≥4.0 cm) (19), hematoma thickness (≥10 mm) (20), ULP development (22) and an elevated CRP level (7.2 mg/dl) (23), have been summarized. The development of retrograde type A aortic dissection (27%) and classic type A aortic dissection (19%) accounts for all fatal complications that can arise as a result of the “wait-and-watch strategy,” and the requirement for additional TEVAR or surgery occurs in as many as 30% of patients during the first 6 months (16, 24). Additionally, Moral et al. found that 10% of IMHB patients, who developed ULP in the acute phase, were correlated with 91% of aorta-related adverse events and 36% mortality (22). Forty-three percent of IMHB patients had TEVAR therapy in the first 2 weeks following diagnosis due to the visibility of an entry rip or aneurysm growth, while 19% of IMHB patients had TEVAR in the first year (25). This procedure is highly sensitive for ruling out classical acute aortic syndrome in patients without low D-dimer levels in the acute phase (26, 27). Additionally, the NLR value reflects the severity of the non-specific inflammatory lesion, which is characterized by an increase in neutrophils and a decrease in lymphocytes. Because an increased NLR may be used to predict worse outcomes and hospital mortality in patients with type A aortic dissection, it is important to note that the NLR value should be measured before treatment begins (28). Furthermore, an increased preoperative NLR ratio may predict early adverse outcomes in patients with uncomplicated type B aortic dissection undergoing TEVAR treatment (29). Overall, further studies are needed to confirm the potential value of D-dimer and NLR in predicting the progression of IMHB patients.

Third, the higher level of eosinophils that remains after the onset of IMHB in DM patients with DPP4i administration may be a key point to explaining why DM patients are not prone to suffering from aortic diseases. The 2019 guidelines for the treatment of DM and cardiovascular disease are described for the first time as the incidence of aortic aneurysm or thoracic aortic dissection in DM patients is lower than tant in non-diabetic patients (1). This phenomenon, which goes against our common sense, was first described by Prakash et al. in 2012 (2). Later, basic research tried to determine how these factors affect the progression of aortic disease, and numerous hypothesized protective effects of DM in aortic aneurysm have been summarized. Many *in vivo* and *in vitro* studies have shown that hyperglycemia, insulin treatment, and different oral antidiabetic drugs can ameliorate the lethal progression of aortic diseases. However, clinical studies have not found the same results and this perspective is still highly controversial (3). DM requires life-long antidiabetic treatment, and long-term administration of oral antidiabetic drugs may mitigate the development of aortic disease. Epidemiological investigations have indicated that the mechanisms resulting in a protective effect of DM on aortic disease development are associated not only with DM pathophysiology but also with antidiabetic treatments. The use of antidiabetic medications was related to a 56% reduction in the abdominal aorta aneurysm growth rate in a trial of 1269 individuals, and this association was independent of confounding variables such as other treatment agents (30). Other studies have also established a negative relationship between metformin administration and aortic aneurysm enlargement and growth (31). Furthermore, a nested case–control study involving 4468 abdominal aorta aneurysm patients and 4468 matched controls found that metformin, sulfonylurea, and thiazolidinedione administration was related to a reduced risk of aneurysm development (32). However, metformin administration was shown to have no significant link to the risk of rupture of abdominal aortic aneurysm (33). In our study, DM patients who received metformin, sulfonylurea, and thiazolidinedione without DPP4i were included in Group B, and remarkably, there were no significant differences in all-cause and aorta-related mortality between Groups A and B during the follow-up period (Table 2 and Figure 2). This finding is similar to that in the report of Kristensen and colleagues (33). The administration of oral antidiabetic drugs has a history of more than 70 years. Why was the protective value of DM first reported in 2012? Sitagliptin, the world’s first commercially available oral DPP4 inhibitor, was first approved for production by the Food and Drug Administration in October 2006. Is it possible that the DPP4i is the key factor that is truly working? As early as 1998, before DPP4i was put on the market, the study of Peterson et al. confirmed that DPP4i could influence the expression of a large number of human chemokines (34, 35). Using DPP4i is associated with an increased plasma level of eotaxin-1, which could result in eosinophil extravasation (35). But until nearly 20 years later, such a “side-effect” of DPP4i had drawn attention from researchers. In a meta-analysis published in 2021, Pan et al. (36) reported that the concentrations of eotaxin-1 in type 2 DM patients were significantly higher than those in control individuals, while no difference in these concentrations was found between prediabetic patients and non-DM patients. Forssmann first reported that DPP4i enhances eotaxin-1 mediated recruitment of eosinophils *in vivo* (37). In 2019, Hollande et al. (12) reported using sitagliptin to recruit eosinophils into tumor tissue, and discovered a distinct mechanism by which the inhibition of DPP4i improves antitumor responses via higher concentrations of the chemokine eotaxin-1 and increased migration of eosinophils into solid tumors. Moreover, a study revealed that eosinophils infiltrating the aortic lesion could release chemokines and play a protective role in abdominal aortic aneurysm progression by regulating macrophage and monocyte polarization and blocking inflammatory process activation in the aortic wall, vascular smooth muscle cells, and endothelial cell dysfunction (13). This may also trigger the normalization of vascular function such as restoring physiological perfusion, maintaining normal oxygenation, and enhancing the angiogenesis process (14). These factors may all contribute to the better clinical outcomes in Group C and explain why this group had the thinnest hematoma thickness even though they had the largest aortic diameter.

Moreover, DPP4i administration probably induces the accumulation of eosinophils by activating axis of interleukin-5, eotaxin-1 and CC-chemokine receptor 3 (CCR3, the eotaxin-1 receptor) axis, which is required for eosinophil accumulation (38). Activation of DPP4-mediated N-terminal truncation may mediate the binding of eotaxin-1 to the chemokine receptor (CCR3) expressed on the surface of eosinophils, and overactivation of DPP4 most likely results in impaired eosinophil chemotaxis (39). In an animal experiment, after DPP4i treatment, there were higher levels of eotaxin-1 and increased concentrations of IL-5 (12). In other animal models, the application of DPP4i could suppress macrophage infiltration and decrease abdominal aortic aneurysm formation (7, 8). We also introduce the role of DPP4i in the recruitment of eosinophils to the aorta in Supplementary Figure 1 (Based on the review of Klion et al. (40) about the recruitment of eosinophils.).

## Limitations

This study has a number of shortcomings due to its restrictions. First, since DM is a lifelong disease, a long-term follow-up is necessary. Estimating the influence of DM and different oral antidiabetic drugs on IMHB progression requires a future longitudinal prospective investigation with multicenter cooperation focusing on more patients and different schemes of oral antidiabetic drugs. Second, since DPP4i administration probably induces the accumulation of eosinophils by resulting in higher concentrations of the chemokine eotaxin-1, further studies with a measurement of eotaxin-1 concentration and expression from blood samples or aortic tissue are required to demonstrate this potential mechanism. Third, in our study, those who required emergency surgery/TEVAR treatment were excluded, and technologies such as TEVAR) and procedures (such as methods of arch repair) definitely affected the final results for patients. It is necessary to conduct additional research to develop a management strategy that is more consistent and uniform to evaluate the influence of these potential risk variables.

## Future directions

DPP4i administration influences IMHB progression and leads to a lower rate of aorta-related adverse events and aorta-related mortality. However, the application of DPP4i alone does not exist in clinical practice, and whether the application of DPP4i could result in the accumulation of eosinophils in the aorta and the specific aortic protective mechanism is still unclear. Therefore, this study requires a series of cell experiments and animal experiments to research the influence of DPP4i on eosinophils and aortic disease, which could provide more evidence to support the potentially protective effect of DPP4i.

## Conclusion

Dipeptidyl peptidase-4 inhibitor administration influences type B intramural hematoma progression in diabetes mellitus patients and leads to a lower aorta-related adverse events rate, aorta-related mortality, and reinterventions than in those who did not receive such drug or those without diabetes mellitus. Variables associated with higher aorta-related adverse events during the follow-up period included a lower eosinophil count and a higher neutrophil to lymphocyte ratio. Furthermore, a lower eosinophil count and a higher neutrophil to lymphocyte ratio were associated with higher aorta-related mortality during the follow-up period. In summary, a higher eosinophil count and a lower neutrophil to lymphocyte ratio are potential protective factors that may explain the potential therapeutic benefit of dipeptidyl peptidase-4 inhibitors.

## Data availability statement

The original contributions presented in this study are included in the article/Supplementary material, further inquiries can be directed to the corresponding author.

## Ethics statement

The studies involving human participants were reviewed and approved by Institutional Ethical Committee of Xiamen University (Xiamen, China). The patients/participants provided their written informed consent to participate in this study.

## Author contributions

QC and ZS: analysis and interpretation. QC and DJ: data collection, writing the article, and statistical analysis. ZS: critical revision of the article. All authors: conception and design, final approval of the article, and agreement to be accountable.

## Supplementary material

SUPPLEMENTARY FIGURE 1 The role of DPP4i in the recruitment of eosinophils to the aorta.

## Abbreviations

CCR3: CC-chemokine receptor 3
CI: confidence interval
CTA: Computed tomography angiography
CRP: C-reactive protein
DDP4i: dipeptidyl peptidase-4 inhibitor
DM: diabetes mellitus
HbA1c: hemoglobin A1c
HR: hazard ratio
IMH: intramural hematoma
IMHB: Type B intramural hematoma
NLR: neutrophil to lymphocyte ratio
OR: Odds ratio
TEVAR: thoracic endovascular aortic repair
ULP: ulcer-like projection.
