# Supplementary material for: The influence of dipeptidyl peptidase-4 inhibitor on the progression of type B intramural hematoma
Source: Front Cardiovasc Med. 2022 Oct 18;9:969357. doi: 10.3389/fcvm.2022.969357 (PMC9623157; doi:10.3389/fcvm.2022.969357)
Supplement: Supplementary file 1 [file Table_1.DOCX]

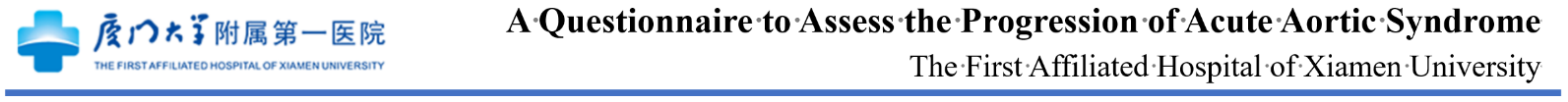


| **Section I: Please Answer Following Questions and Provide General Information** |
| --- |
| 1.1 Name: ____________________________________ Age: __________ Gender: Male □ Female □ |
| 1.2 Address: _________________________________ City: ____________ Zip Code: ___________ |
| 1.3 Home phone: _______________________ Work phone: ____________ Cell phone: ____________ |
| 1.4 Your primary cardiac surgeon’s name: ___________________________________________________ |
| 1.5 What is your nationality and religion? ¨  Han □ Manchu □ Mongolian □ Hui □ Tibetan □ Others □: ______________  Buddhism □ Taoism □ Christianity □ Islam □ others □: ______________ |
| 1.6 Height and Weight: _____cm _____kg |
| 1.7 Date of filling: ___________ |
|  |
| **Section II: Socioeconomic/Family Medical History** |
| 2.1. Marital status: Single □ Married □ Divorced □ Widowed □ |
| 2.2 How many people live in your household? _________ |
| 2.3 Does anyone else who lives with you have acute aortic syndrome? No □ Yes (Who?): _________________ |
| 2.4 Does anyone else who lives with you have thoracic/abdominal aneurysm? No □ Yes (Who?): _________________ |
| 2.5 Occupation: ____________________________________; Work hours: ____________________ |
| 2.6 Smoking □ Drinking □ Drug abuse □ |
| 2.6.1 Do you smoke cigarettes? If yes, how many cigarettes a day? _______ |
| 2.6.2 How many years have you been smoking? _______ |
| 2.6.3 Have you ever been enrolled in a tobacco cessation program? Yes □ No □ |
| 2.6.4 Does anyone in your house smoke? Yes □ No □ |
| 2.7 Last grade of school completed: Middle school □ High school □ College □ |
| 2.8 What type of physical activity do you currently do?  Aerobic Workout Bicycling □ Running/Jogging Swimming □ Walking □ None □ |
| 2.9 How often do you do physical activity?  1-3 times a week □ 3-5 times a week □ 5-7 times a week □ inconsistently □ none□ |
|  |
| **Section III: History of Chronic Diseases and Management** |
| **3.1 Hypertension** |
| 3.1.1 What prescription medications do you take? Please list: |
| 3.1.2 Do you take non-prescription medications or supplements (for example, aspirin, vitamins, etc.)? Yes □ No □  If yes, please list: |
| 3.1.3 Are you having any problems taking your medications? Yes □ No □  If yes, please explain: |
| 3.1.4 What was your last systolic blood pressure reading? (Top number) _____ mmHg |
| 3.1.5 Your last diastolic blood pressure reading? (Bottom number) _____ mmHg |
| 3.1.6 Have you had a blood pressure reading of 140/90 or less in the last year? Yes □ No □ |
| 3.1.7 Which of the following symptoms have you had?  Blurry □ Vision □ Chest Pain □ Dizziness □ Headaches □ None □ Other: |
| **3.2** **Dyslipidemia** |
| 3.2.1 Your last test result of triglyceride: _____ (mmol/L) |
| 3.2.2 Your last test result of total cholesterol: _____ (mmol/L) |
| 3.2.3 Your last test result of high-density lipoprotein cholesterol: _____ (mmol/L) |
| 3.2.4 Your last test result of low-density lipoprotein cholesterol: _____ (mmol/L) |
| 3.2.5 Your last test result of Apolipoprotein α1_____ or Apolipoprotein β_____ |
| **3.3 Coronary Heart Disease** |
| 3.3.1 Had any non-surgical or surgical procedures for the treatment of coronary heart disease? Yes □ No □  If yes, indicate the non-surgical □ or surgical □ |
| 3.3.2 Percutaneous coronary intervention (PCI) (angioplasty)  Date of treatment: ________________________________ Date of admission: __________________________  Indicate treatment facility: ____________________________________________________________________  Indicate the condition that resulted in the need for the procedure/treatment: __________________________  Date of discharge: ________________________________ |
| 3.3.3 Coronary artery bypass surgery  Date of treatment: ________________________________ Date of admission: __________________________  Indicate treatment facility: ____________________________________________________________________  Indicate the condition that resulted in the need for the procedure/treatment: __________________________  Date of discharge: ________________________________ |
| 3.3.4 Cardiac/Heart transplants  Date of treatment: ________________________________ Date of admission: __________________________  Indicate treatment facility: ____________________________________________________________________  Indicate the condition that resulted in the need for the procedure/treatment: __________________________  Date of discharge: ________________________________ |
| 3.3.5 Implanted cardiac pacemaker  Date of treatment: ________________________________ Date of admission: __________________________  Indicate treatment facility: ____________________________________________________________________  Indicate the condition that resulted in the need for the procedure/treatment: __________________________  Date of discharge: ________________________________ |
| 3.3.6 Automatic implantable cardioverter defibrillator (AICD)  Date of treatment: ________________________________ Date of admission: __________________________  Indicate treatment facility: ____________________________________________________________________  Indicate the condition that resulted in the need for the procedure/treatment: __________________________  Date of discharge: ________________________________ |
| 3.3.7 Ventricular aneurysmectomy  Date of treatment: ________________________________ Date of admission: __________________________  Indicate treatment facility: ____________________________________________________________________  Indicate the condition that resulted in the need for the procedure/treatment: __________________________  Date of discharge: ________________________________ |
| 3.3.8 Other surgical and/or non-surgical procedures for the treatment of a heart condition, describe: ________________  _______________________________________________________________________________________________ |
| **3.4 Obstructive Sleep Apnea** |
| 3.4.1 Snoring?  Do you Snore Loudly (loud enough to be heard through closed doors or your bed-partner elbows you for snoring at night)? Yes □ No □ |
| 3.4.2 Tired?  Do you often feel Tired, Fatigued, or Sleepy during the daytime (such as falling asleep during driving or talking to |
| someone)? Yes □ No □ |
| 3.4.3 Observed?  Has anyone Observed you Stop Breathing or Choking/Gasping during your sleep? Yes □ No □ |
| 3.4.4 Pressure?  Do you have or are being treated for High Blood Pressure? Yes □ No □ |
| 3.4.5 Body Mass Index more than 35 kg/m2? Yes □ No □ |
| 3.4.6 Age older than 50? Yes □ No □ |
| 3.4.7 Neck size large? (Measured around Adams apple)  Is your shirt collar 40cm or larger? Yes □ No □ |
| 3.4.8 Had received any polysomnography examination? Yes □ No □ |
| 3.4.9 Had received any obstructive sleep apnea treatment? Yes □ No □  Which treatment? CPAP □ Anatomical interventions □ Oral appliance □ Sleep consolidation aid □ |
| 3.4.1 Severity of obstructive sleep apnea. Severe □ Medium □ Wild □ |
| **3.5 Renal Failure** |
| 3.5.1 When did you begin or restart your hemodialysis treatment? Yes □ No □ Beginning data: ___________ |
| 3.5.2 Have you ever had chronic peritoneal dialysis treatment? Yes □ No □ Beginning data: ___________ |
| 3.5.3 Have you had a kidney transplant? Yes □ No □ Operation data: ___________ |
| **3.6 Stroke** |
| 3.6.1 Is your blood pressure greater than 120/80 mm/Hg? Yes □ No |
| 3.6.2 Is your fasting blood sugar greater than 100 mg/dL? Yes □ No |
| 3.6.3 Have you been diagnosed with atrial fibrillation? Yes □ No |
| 3.6.4 Is your body mass index greater than 25kg/m^2^? Yes □ No |
| 3.6.5 Is your diet high in saturated fat, trans fat, sweetened beverages, salt, excess calories? Yes □ No |
| 3.6.6 Is your total blood cholesterol greater than 180 mg/dL? Yes □ No |
| 3.6.7 Do you participate in 40 minutes of moderate to vigorous physical activity 3-4 days a week? Yes □ No |
| 3.6.8 Do you have a family history of stroke? Yes □ No |
| **3.7 Peripheral ischemia diseases** |
| 3.7.1 Do you experience aching, cramping or pain in your arms, legs, thighs or buttocks when you walk or exercise? Yes □ No □ |
| 3.7.2 If you answered yes to the first question, does the pain subside with rest? Yes □ No □ |
| 3.7.3 Do you have numbness and tingling in the arms or lower legs? Yes □ No □ |
| 3.7.4 Are your fingers or toes pale, discolored or bluish? Yes □ No □ |
| 3.7.5 Are your hands or feet cold to the touch? Yes □ No □ |
| 3.7.6 Do you have any sores or ulcers on your legs or feet that don't heal? Yes □ No □ |
| 3.7.7 Do you have difficult to control blood pressure and are on more than 2 blood pressure medications? Yes □ No □ |
| 3.7.8 Have you ever experienced a stroke, ministroke or transient ischemic attack (TIA)? Yes □ No □ |
| **3.8 Diabetes Mellitus** |
| 3.8.1 How long have you had diabetes or year diagnosed? ___________________________________ |
| 3.8.2. What type of diabetes do you have? Type 1 □ Type 2 □ |
| 3.8.3 Chronic Complications: Are you aware of or have you ever been told by a doctor you have any of these problems? Please rate as L=Little M=Moderate S=Severe  Eye problems: L □ M □ S □ Heart/artery problems: L □ M □ S □ Nerve problems: L □ M □ S □  Teeth/gums problems: L □ M □ S □ Feet/leg problems, explain: L □ M □ S □  Skin problems: L □ M □ S □ GI problems: L □ M □ S □ Sexual problems, explain: L □ M □ S □  Kidney problems: L □ M □ S □ Frequent infections: L □ M □ S □  Other problems, explain: |
| 3.8.4 Do you test your blood for sugar? Yes □ No □ |
| 3.8.5 How often do you test?  Once a day □ 2 or more times a day □ Once/Twice a week □ |
| 3.8.6 How often do you have HIGH blood sugar? (250 or more)  Daily □ Several times a week □ A few times a month □ Once in a while □ |
| 3.8.7 How often do you have LOW blood sugar (70 or less)?  Daily □ Several times a week □ A few times a month □ Once in a while □ |
| 3.8.8 Do you have access to your antidiabetic drugs?  No □  Yes □:  Short-acting insulins: □ Drug Name: _______Dosage: ______Date of admission: ______________  Intermediate-acting insulins: □ Drug Name: ______Dosage: _________ Date of admission: _______  long-acting insulins: □ Drug Name: _____Dosage: ________ Date of admission: _______  Biguanides: □ Drug Name: _____________Dosage: _________ Date of admission: _______  Thiazolidinediones: □ Drug Name: _______Dosage: _________ Date of admission: _______  Sulfonylureas: □ Drug Name: ________Dosage: _____________ Date of admission: _______  Nonsulfonylurea secretagogues: □ Drug Name: ________Dosage: _____ Date of admission: _______  Alpha-glucosidase inhibitors: □ Drug Name: _______Dosage: ________ Date of admission: _______  Glucagon-like peptide: □ Drug Name: _______Dosage: ________ Date of admission: _______  Dipeptidyl peptidase-4 inhibitors: □ Drug Name: _______Dosage: _______ Date of admission: _______ |
| **Section IV: Acute Aortic Syndrome** |
| 4.1 Acute aortic syndrome calcification:  Aortic dissection □ Penetrating aortic ulcer □ Intramural Hematoma □ |
| 4.2 Stanford System: Type A □ Type B □ |
| 4.3 Other aortic diseases: Aortic aneurysm□ Aortic coarctation □ Traumatic aortic damage □  Aortic valvular diseases □ Connective tissue disease □ |
| 4.4 Complicated or uncomplicated acute aortic syndrome? The definition of “uncomplicated” depended on the syndrome after medical treatment (without signs of aortic rupture [uncontrollable chest/back pain combined with a precipitous decrease in blood pressure] on admission and controllable syndromes) and radiographic findings from the CTA examination on admission (without periaortic hematoma and ulcer-like projection).  Complicated □ Uncomplicated □ |
| 4.5 Lethal organ ischemia on admission: Yes □ No □ |
| 4.6 Emergency surgical or interventional therapy: Yes □ No □ |
| 4.7 Decline any medical or further treatment on admission: Yes □ No □ |
| 4.8.1 Antihypertension medical treatment  ACEI/ARB □ Drug Name: _______Dosage: ______Date of admission: ______________  β-blockers □ Drug Name: _______Dosage: ______Date of admission: ______________  Calcium antagonists □ Drug Name: _______Dosage: ______Date of admission: ______________  Diuretic □ Drug Name: _______Dosage: ______Date of admission: ______________  Urapidil □ Drug Name: _______Dosage: ______Date of admission: ______________  Nitrates □ Drug Name: _______Dosage: ______Date of admission: ______________ |
| 4.8.2 Anticoagulant & antiplatelet treatment  Heparin & low molecular heparin □ Drug Name: _______Dosage: ______Date of admission: ____________  Warfarin □ Drug Name: _______Dosage: ______Date of admission: ______________  Aspirin □ Drug Name: _______Dosage: ______Date of admission: ______________  P2Y12 inhibitors □ Drug Name: _______Dosage: ______Date of admission: ______________  Novel Oral Anticoagulants □ Drug Name: _______Dosage: ______Date of admission: ______________ |
| 4.8.3 Lipid-lowering drugs  Statins □ Drug Name: _______Dosage: ______Date of admission: ______________  Cholesterol absorption inhibitors □ Drug Name: _______Dosage: ______Date of admission: ______________  Bebutyric acid drugs □ Drug Name: _______Dosage: ______Date of admission: ______________ |
| 4.8.4 Aorta measurement on admission  Diameter of ascending aorta: _______ (mm) Diameter of descending aorta: _______ (mm)  Hematoma thickness: _______ (mm) |
| 4.8.5 Aorta measurement on day 14  Diameter of ascending aorta: _______ (mm) Diameter of descending aorta: _______ (mm)  Hematoma thickness: _______ (mm) |
| 4.8.6 Aorta measurement before discharge  Diameter of ascending aorta: _______ (mm) Diameter of descending aorta: _______ (mm)  Hematoma thickness: _______ (mm) |
| 4.8.7 Aorta-related adverse events during the acute phase Yes □ No □  Increased pleural effusion Yes □ No □  Hematoma thickening (thickness ≥10 mm) Yes □ No □  Development of ulcer-like projection Yes □ No □  Development of aortic dissection Yes □ No □  Aortic aneurysm/pseudoaneurysm development Yes □ No □ |
| 4.8.8 Emergency interventions or treatment after 14 days  Surgery: Date of treatment: __________________________ Date of admission: ________________________  Indicate treatment facility: ____________________________________________________________  Indicate the condition that resulted in the need for the procedure/treatment: _______________________  Date of discharge: ________________________________  TEVAR: Date of treatment: __________________________ Date of admission: __________________________  Indicate treatment facility: ____________________________________________________________  Indicate the condition that resulted in the need for the procedure/treatment: _______________________  Date of discharge: ________________________________  Supplier of stent:  □ TAG (W.L Gore & Associates, Flagstaff, Ariz); □Zenith TX2 (Cook, Inc, Bloomington, Ind);  □ Valiant (Medtronic, Inc, Minneapolis, Minn); □Ankura (Lifetechmed, Inc, Shenzhen, China)  Died after Surgery/TEVAR Yes □ No □ |
| 4.8.9 Progression of acute aortic syndrome (intramural hematoma)  Stable/resolution of hematoma Yes □ No □ Date: _____  Aorta-related adverse events during the follow-up period Yes □ No □ Date: _____  Hematoma thickening (thickness ≥10 mm) Yes □ No □ Date: _____  Development of ULPs Yes □ No □ Date: _____  Development of aortic dissection Yes □ No □ Date: _____  Aortic aneurysm/pseudoaneurysm Yes □ No □ Date: _____  Reintervention Yes □ No □ Date: _____ Surgery: □ TEVAR: □  All-cause death cases Yes □ No □  Aorta-related death cases Yes □ No □ Date: _____  Non-aorta-related death case Yes □ No □ Date: _____ |
| 4.8.10 Progression of acute aortic syndrome (aortic dissection/PAU)  Stable or thrombus of aortic dissection Yes □ No □  Aorta-related adverse events during the follow-up period Yes □ No □ Date: _____  False lumen enlarges Yes □ No □ Date: _____  Development of type A aortic dissection Yes □ No □ Date: _____  Aortic aneurysm/pseudoaneurysm Yes □ No □ Date: _____  Reintervention Yes □ No □ Date: _____ Surgery: □ TEVAR: □  All-cause death cases Yes □ No □  Aorta-related death cases Yes □ No □ Date: _____  Non-aorta-related death case Yes □ No □ Date: _____ |
| 4.8.11 Laboratory test results (On admission, Day 14, Before discharge, Follow-up)  White blood cell: 1) ________2) ________3) ________4) ________  Neutrophils: 1) ________2) ________3) ________4) ________  Lymphocyte: 1) ________2) ________3) ________4) ________  Eosinophils: 1) ________2) ________3) ________4) ________  Basophils: 1) ________2) ________3) ________4) ________  Monocytes: 1) ________2) ________3) ________4) ________  Platelet: 1) ________2) ________3) ________4) ________  C-reactive protein: 1) ________2) ________3) ________4) ________  D-dimer: 1) ________2) ________3) ________4) ________  Blood glucose: 1) ________2) ________3) ________4) ________  HbA1c: 1) ________2) ________3) ________4) ________  Creatinine: 1) ________2) ________3) ________4) ________  Matrix metalloproteinase 2: 1) ________2) ________3) ________4) ________  Matrix metalloproteinase 9: 1) ________2) ________3) ________4) ________ |
| 4.8.12 Regular CTA reexamination  Yes □ Date: _____ Results: _____  No □ Reasons: _____ |
|  |
| **Supplement I: Additional Information** |
| S1. Would you like to participate in our acute aortic syndrome evaluation program? (This a free benefit that is offered by the first affiliated hospital of Xiamen University) Yes □ No □ |
| S2. No classes or travel are required. (A nurse will call you on the telephone) Yes □ No □ |
| S3 What days are best to call you? Mon □ Tue □ Wed □ Thu □ Fri □ Any Day □ |
| S4 What are the best times to call you?  7-9 am 9-11 am □ 1-3 pm 3-5 pm □ 11 am-1 pm □ |
|  |
| **Supplement II:** Examiner's Certification and Signature |
| S5. Examiner's signature:  Date: |
| S6. Examiner's printed name and title (e.g., MD, PhD, Mr., Miss): |
| S7. Examiner's phone/fax numbers: |
| S8. Examiner's address: |
