# Supplementary material for: The influence of dipeptidyl peptidase-4 inhibitor on the progression of type B intramural hematoma
Source: Front Cardiovasc Med. 2022 Oct 18;9:969357. doi: 10.3389/fcvm.2022.969357 (PMC9623157; doi:10.3389/fcvm.2022.969357)
Supplement: Supplementary file 2 [file Table_2.DOCX]

| **Supplement 2 The Independent-Samples Kruskal-Wallis Test and Pairwise Comparisons of Three Groups** | | | | | | | | |
| --- | --- | --- | --- | --- | --- | --- | --- | --- |
| **Variables** | **Independent-Samples Kruskal-Wallis Test** | | **Pairwise Comparisons of Three Groups** | | | | | |
|  |  |  | **Group A and Group B** | | **Group A and Group C** | | **Group B and Group C** | |
|  | **Test Statistic** | ***P* Value** | **Test Statistic** | **Adj. *P* Value^a^** | **Test Statistic** | **Adj. *P* Value^a^** | **Test Statistic** | **Adj. *P* Value^a^** |
| **White blood cell (10^9/L)** | 37.436 | <0.001 | 8.734 | 0.629 | 40.484 | <0.001 | 31.750 | <0.001 |
| **Neutrophils (10^9/L)** | 49.492 | <0.001 | 14.141 | 0.127 | 47.688 | <0.001 | 33.547 | <0.001 |
| **Lymphocyte (10^9/L)** | 51.170 | <0.001 | -18.891 | 0.020 | -49.359 | <0.001 | -30.469 | <0.001 |
| **Eosinophils (10^9/L)** | 67.630 | <0.001 | -40.906 | <0.001 | -55.094 | <0.001 | -14.187 | 0.041 |
| **C-reactive protein (mg/L)** | 36.527 | <0.001 | 36.094 | <0.001 | 36.797 | <0.001 | 0.703 | 1.000 |
| **D-dimer (ug/mL)** | 63.996 | <0.001 | 21.438 | 0.006 | 55.250 | <0.001 | 33.813 | <0.001 |
| **Neutrophil to lymphocyte ratio** | 65.615 | <0.001 | 26.859 | <0.001 | 56.391 | <0.001 | 29.531 | <0.001 |
| **Diameter of ascending aorta (mm)** | 17.692 | <0.001 | -4.891 | 1.000 | -27.453 | <0.001 | -22.562 | 0.004 |
| **Diameter of descending aorta (mm)** | 13.417 | 0.001 | -5.891 | 1.000 | -24.437 | 0.001 | -18.547 | 0.023 |
| **Hematoma thickness (mm)** | 25.653 | <0.001 | 12.969 | 0.188 | 34.891 | <0.001 | 21.922 | 0.005 |

^a^: The significance values were adjusted by the Bonferroni correction for multiple tests and gained the adjusted *P* values.
