# Supplementary material for: The influence of dipeptidyl peptidase-4 inhibitor on the progression of type B intramural hematoma
Source: Front Cardiovasc Med. 2022 Oct 18;9:969357. doi: 10.3389/fcvm.2022.969357 (PMC9623157; doi:10.3389/fcvm.2022.969357)
Supplement: Supplementary file 3 [file Table_3.DOCX]

| **Supplement 3 The Results of the Z-Test That Compared Each Column Proportions Among Different Groups** | | | | |
| --- | --- | --- | --- | --- |
| **Variables** | **Group A**  **(n=32)** | **Group B**  **(n=32)** | **Group C**  **(n=32)** | ***P* value** |
| **Aorta-related adverse events during the acute phase, n (%)** | 11 (34.4%) ^a^ | 8 (8.3%) ^a^ | 1 (3.1%) ^b^ | 0.007 |
| **Development of ULPs, n (%)** | 9 (28.1%) ^a^ | 6 (18.8%) ^a b^ | 1 (3.1%) ^b^ | 0.025 |
| **TEVAR, n (%)** | 10 (31.3%) ^a^ | 7 (21.9%) ^a b^ | 1 (3.1%) ^b^ | 0.013^c^ |
| **Aorta-related adverse events during the follow-up period, n (%)** | 13 (40.6%) ^a^ | 8 (25.0%) ^a^ | 1 (3.1%) ^b^ | 0.002 |
| **Development of ULPs during the follow-up period, n (%)** | 9 (28.1%) ^a^ | 5 (15.6%) ^a b^ | 1 (3.1%) ^b^ | 0.023 |
| **Reintervention during the follow-up period, n (%)** | 13 (40.6%) ^a^ | 8 (25.0%) ^a^ | 1 (3.1%) ^b^ | 0.002 |
| **All-cause death cases during the follow-up period, n (%)** | 10 (31.3%) ^a^ | 4 (12.5%) ^a b^ | 1 (3.1%) ^b^ | 0.007 |
| **Aorta-related death cases, during the follow-up period, n (%)** | 8 (25.0%) ^a^ | 3 (9.4%) ^a b^ | 0 (0.0%) ^b^ | 0.007 |

TEVAR, thoracic endovascular aortic repair; ULP: Ulcer-like projection.

**^a b c^:** Different subscript letter denotes a subset of group categories whose column proportions differ significantly from each other at the 0.05 level.
