# Supplementary figures and images for: The influence of dipeptidyl peptidase-4 inhibitor on the progression of type B intramural hematoma
Source: Front Cardiovasc Med. 2022 Oct 18;9:969357. doi: 10.3389/fcvm.2022.969357 (PMC9623157; doi:10.3389/fcvm.2022.969357)

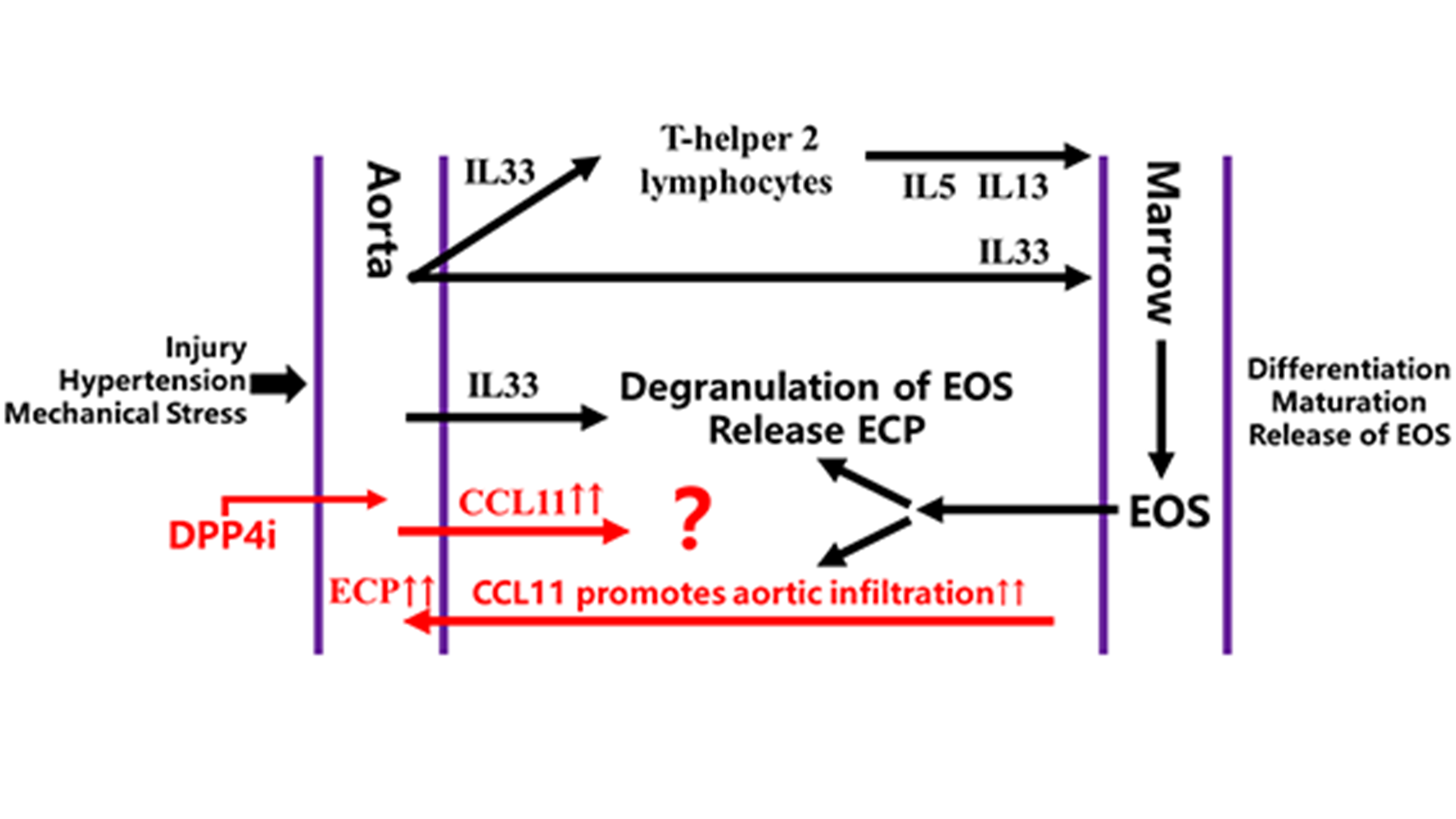

Supplement: SUPPLEMENTARY FIGURE 1 — The role of DPP4i in the recruitment of eosinophils to the aorta. [file Image_1.TIF]
